# Clinical Management of Facemasks for Early Treatment of Class III Malocclusion: A Survey among SIDO Members

**DOI:** 10.3390/dj12070207

**Published:** 2024-07-05

**Authors:** Lorenzo Franchi, Michele Nieri, Patrizia Marti, Annamaria Recupero, Alessandra Volpe, Alessandro Vichi, Cecilia Goracci

**Affiliations:** 1 Department of Experimental and Clinical Medicine, University of Florence, 50121 Florence, Italy; michele.nieri@unifi.it; 2 Santa Chiara Fab Lab, Department of Social Political and Cognitive Sciences, University of Siena, 53100 Siena, Italy; patrizia.marti@unisi.it (P.M.); annamaria.recupero@unisi.it (A.R.); 3 Department of Medical Biotechnologies, University of Siena, 53100 Siena, Italy; a.volpe7@student.unisi.it (A.V.); cecilia.goracci@unisi.it (C.G.); 4 Dental Academy, University of Portsmouth, Portsmouth PO1 2UP, UK; alessandro.vichi@port.ac.uk

**Keywords:** Class III malocclusion, facemask, survey

## Abstract

To evaluate whether there are differences among orthodontists in the clinical management of facemask treatment for early treatment of Class III malocclusion, a survey consisting of 16 questions was conducted among members of the Italian Society of Orthodontics (SIDO). Sixty percent of the respondents were Specialists in Orthodontics (S) whereas 40% were General Dentists practicing Orthodontics (GD). Descriptive statistics were calculated to summarize the collected data. Differences in answers between S and GD were assessed with the Fisher’s exact test for dichotomous variables, chi-square test for qualitative variables, and Mann–Whitney test for ordinal variables. A total of 151 clinicians participated in this survey. As for treatment timing, about 80% of the participants reported treating Class III patients with RPE and facemask between 5 and 8 years of age. Most of the participants requested the patients to wear the facemask in the afternoon and at night for a period of 9 or 12 months with recommended forces of 500 g per side. Comparisons between S and GD showed that S preferred the Petit facemask whereas GD favored the Delaire’s type facemask (Fisher’s Exact test, *p* = 0.0005). S and GD also differed significantly in their judgment of the most critical time of treatment, which for the majority of GD was the initial period but for the S was the final period (Chi-square test *p* = 0.0188). This survey showed that the facemask is not well received by the patients who, along with their parents, express concerns regarding its tolerability.

## 1. Introduction

Class III malocclusion is regarded as a complex maxillofacial disorder [1]. Although several appliances have been utilized in the early treatment of Class III malocclusions (facemask, chin cup, tandem traction bow appliance, mandibular headgear, etc.) facemask therapy remains the most commonly used approach [2,3]. Facemask treatment during the early developmental phases, when the circum-maxillary sutures are still immature [4,5,6], generally before age 10–11 years [2,7], has been advocated to enhance the contribution of the orthopedic effect in the overall management of Class III malocclusion, to improve dental occlusion and facial aesthetics, and to reduce the need for future orthognathic surgery [1]. Evidence has been collected that early facemask therapy is effective at improving the maxilla–mandibular relationship in Class III malocclusions by enhancing forward growth of the maxilla and limiting mandibular growth [1,2,3,8,9].

Currently, available facemasks consist of a quadrangular metal framework (Delaire type) or of a single midline stainless steel rod (Petit type) to which a forehead and a chin pad are connected. It has been pointed out [10] that the Delaire facemask offers good rigidity but is rather bulky and can cause difficulties with side-sleeping and wearing eyeglasses. On the contrary, the Petit type facemask is simple and comfortable for wearing at night and for those children wearing eyeglasses, but it can easily dislodge and sometimes get cockeyed [10]. In order to apply a forward traction to the maxilla, heavy elastics, transmitting a force as high as 500 g per side [11,12], are attached from the intraoral anchorage system, which is most often provided by a rapid palatal expander (RPE), to a cross bar extending in front of the mouth. Frontal and mental chin pads of the commercially available facemasks are made of hard acrylic resin, lined with a soft closed-cell foam on the side contacting the skin. Forehead and chin pads are only available in standardized shapes and in two sizes.

It has clearly emerged that the success of treatment with the facemask heavily depends on patient’s collaboration with the requested wearing time, which can range between 14 and 24 h a day, depending on the practitioner’s recommendation [13]. Commercially available facemasks are found by the patient to be unaesthetic and uncomfortable [13,14]. In a survey assessing the acceptability and attractiveness of intra- and extra-oral orthodontic appliances, the facemask was rated as the least acceptable device [14]. In addition to poor aesthetics, children complain that facemasks are bulky and unstable, frequently causing skin irritations, particularly on the chin [15,16]. Moreover, the unintentional repeated dislocation of a poorly fitting standard chin cup may produce a gingival recession of the lower incisors [17].

Several attempts have been made to customize frontal and mental chin pads for increased fit and patient comfort [15,18,19,20]. Some of these procedures have involved taking a plaster [19] or an alginate [20] impression of the patient’s face, which can be an unpleasant experience for the child. Cacciatore et al. [18] used a putty-consistency polyvinyl siloxane to reline the acrylic resin chin pad of the standard marketed facemask, whereas Ierardo et al. [15] proposed to insert underneath the mental chin pad a 3-mm thick layer of soft-bite material thermoformed on a plaster model of the patient’s chin.

Efforts have been made also to monitor patients’ compliance with wearing the facemask [13,21,22]. However, Arreghini et al. [21] reported that patients’ awareness of being monitored did not result in full adherence to the orthodontist’s recommendations.

It appeared interesting, therefore, to explore whether differences exist among orthodontists in the clinical management of facemask treatment and to collect information on how clinicians handle the critical issues of patient’s acceptance of the device and compliance with the recommended wear time. For this purpose, a survey was conducted among members of the Italian Society of Orthodontics (SIDO) as a representative sample of orthodontic practitioners. In particular, a comparison between General Dentists practicing Orthodontics (GD) and Specialists in Orthodontics (S) will be performed.

## 2. Materials and Methods

This paper was written following the CROSS checklist [23] that is the guideline that is recommended for designing and conducting survey studies.

### 2.1. Study Design

A web-based questionnaire regarding the clinical management of maxillary facemasks was developed in 2020 and approved by the Board Council of the Italian Society of Orthodontics (SIDO) on 3 July 2020 (Table 1).

The targets of this cross-sectional survey were the members of the Italian Society of Orthodontics (SIDO), who in July 2020 received by email a link to access online the questionnaire written in Italian. In May 2021, a second reminder was sent with the recommendation not to participate again to those who had already submitted their replies. All recorded data were treated anonymously by means of the SurveyMonkey web-based software (https://www.surveymonkey.com/) (accessed on 28 May 2021). SIDO members consist of both General Dentists practicing Orthodontics (GD) and Specialists in Orthodontics (S).

In the first page of the survey was reported what were the addressees and the objectives of this study (Table 1). The first question was meant to collect the respondent’s informed consent to participate. The subsequent question aimed to classify the respondents as GD or S, with the intention of comparing the replies between these two groups. Questions 3–7 collected information on patient’s age at the start of facemask therapy or the type of facemask used, recommended wear time, usual treatment duration, and applied force. Questions 8–10 investigated what patients and parents reported to the SIDO member about facemask therapy. Additionally, the clinician’s opinion about the most critical time during facemask treatment was ascertained in question 11. Questions 12–15 collected information on any occurrence that was possibly challenging the patient’s compliance and reducing the wear time. The last question surveyed the methods adopted by parents to increase their child’s cooperation.

### 2.2. Statistical Analysis

Descriptive statistics were calculated to summarize the collected data. Frequency and percentage were calculated for qualitative variables whereas median and interquartile interval were calculated for ordinal variables. The statistical significance of the differences in the provided answers between S and GD was assessed for the questions that required a direct judgment from the clinicians (questions 3 through 7, question 11, and questions 14 through 16) with the Fisher’s exact test for dichotomous variables, the chi-square test for qualitative variables, and the Mann–Whitney test for ordinal variables. The significance level was set at *p* < 0.05. The statistical calculations were handled by statistical software (JMP version 13.0.0, SAS Institute Inc, Cary, NC, USA).

## 3. Results

A total of 151 questionnaires were returned by the end of June 2021 (response rate 5% out of 2997 SIDO members). The percentage of specialists in Orthodontics among the respondents was 60% (Table 1, question 2).

Seventy-nine percent of the participants reported treating Class III patients with facemask most frequently between 5 and 8 years of age, 20% after 8 years of age, and only 1% at an age earlier than 5 years (Table 1, question 3). A slight preference was observed for the Delaire facemask (52%) over the Petit type, which was the device chosen by 48% of the clinicians (Table 1 question 4). The majority of participants requested that the patients wear the facemask at night and in the afternoon or for as long as possible when at home for a period of 9 or 12 months (Table 1, questions 4 and 5). Regarding the amount of elastic force, 60% of the respondents applied 500 g per side, 22% applied a lighter force, and 18% applied a heavier force (Table 1, question 7).

Question 11 revealed that for the majority of the clinicians the most critical time for compliance with therapy was the initial period right after facemask delivery (54%), when the patient can experience low tolerance for the appliance or have a troublesome adaptation to it.

Questions 12 and 13 focused on the problems referred to by patients and parents during facemask therapy. Skin irritations were the most frequent adverse events according to both patients and parents. Less frequently observed complications were lower lip or mouth-corner lesions. Facemask application was rarely deemed painful, though it was most often considered uncomfortable. Neither patients nor parents frequently complained about the rigidity or aesthetics of the device. Difficulty in falling asleep or nocturnal awakenings were not frequent, whereas both parents and patients witnessed appliance displacements from the correct position with a child’s movements during sleep. The facemask was not usually found difficult to apply or to keep clean.

Overall, the majority of the clinicians did not notice the development of gingival recessions of the lower incisors site due to pressure from the chin pad (Table 1, question 14).

When asked about the factors possibly hampering the success of facemask treatment, the practitioners indicated patients’ and parents’ collaboration followed by the inability to monitor wear time and the adequacy of applied forces (Table 1, question 15).

To stimulate patients’ compliance with the therapy, clinicians mainly explain to the parents what the therapy consists of and what results are expected, thereby motivating them to obtain or stimulate child compliance. On the contrary, it is not common among the clinicians to propose a reward strategy for good cooperation (Table 1, question 16).

Comparisons between General Dentists practicing Orthodontics (GD) and Specialists in Orthodontics (S) are reported in Table 2, Table 3, Table 4, Table 5 and Table 6. When comparing the answers provided by S and GD, a statistically significant difference emerged in the choice of the facemask design, with S preferring the Petit’s type and GD favoring the Delaire’s type (Fisher’s Exact test, *p* = 0.0005) (Table 2). S and GD also differed significantly in their judgment of the most critical time of treatment, which for the majority of GD was the initial period whereas for the S was the final period (Chi-square test *p* = 0.0188) (Table 3). As for questions 14 through 16, no statistically significant differences were found between S and GD (Table 4, Table 5 and Table 6).

## 4. Discussion

The purpose of this study was to conduct a survey among members of the Italian Society of Orthodontics (SIDO) as a representative sample of orthodontic practitioners. In particular, it appeared to be interesting to evaluate the clinical management of facemask treatments among orthodontists and to collect information on how clinicians handle the critical issues of patient’s acceptance of the device and compliance with the recommended wear time. Additionally, comparisons between General Dentists practicing Orthodontics (GD) and Specialists in Orthodontics (S) were performed for some of the questions that were in the survey.

As for treatment timing, about 80% of the participants reported treating Class III patients with RPE and a facemask between 5 and 8 years of age as suggested by the Italian Clinical Recommendations in Odontostomatology [24]. The respondents indicated equal preferences for either Delaire or Petit facemasks in everyday clinical practice. Most of the participants requested that the patients wear the facemask in the afternoon and at night for a period of 9 or 12 months (Table 1, questions 4 and 5). This result corresponded to the time duration for wearing the facemask that is recommended by clinicians [13,21]. Regarding the amount of the elastic force, the majority of the respondents applied the recommended forces (500 g per side) [11,12].

Interestingly, despite the fact that parents consider the facemask to be an appliance that is needed for the therapy = that will give the desired results, from questions 9 and 10 it emerged that both parents and patients were worried about duration of facemask treatment. This result is not surprising at all if we consider that it has been clearly shown that commercially available facemasks are found by the patient to be unaesthetic and uncomfortable [13,14]. Additionally, the majority of the clinicians reported that the most critical time for compliance with therapy was the initial period immediately after facemask delivery, when the patient typically experiences difficulties in adaptation to any orthodontic appliance [21].

Probably the most interesting results of this survey come from the results of the last four questions that clearly highlight the fact that the use of the facemask can be associated with complications that may jeopardize patients’ collaboration and, therefore, the success of the treatment.

It has been reported in the literature [13,14,15,16,17,18,21,22] and noted in the clinical practice that facemask treatment is often disrupted by the occurrence of complications, mainly attributed to bad fit or to bulkiness of the components. One of the objectives of the present survey was to verify how broadly this experience was shared in a sample of respondents that is representative of the Italian community of orthodontists. This study confirmed that although before starting treatment parents are mainly concerned with the painfulness of the appliance, or with its possible interference with the daily activities and sleep habits of the child, the most common and serious issue reported during facemask therapy is the occurrence of skin irritation phenomena. Additionally, according to the surveyed orthodontists, skin reactions due to the pressure from the chin rest are more frequent than are other undesired effects such as forehead skin irritations or lesions of the lower lip mucosa. In the study by Kim et al. [16], skin reactions at the chin level were observed in 43.5% of the children treated with the Delaire-type facemask. The lesions were classified as frictional contact dermatitis and were ascribed to low-grade frictional trauma. Also Gazzani et al. [25], who found adverse effects on facial skin to be very frequent in daily practice using a finite element analysis, pointed out that either type of marketed facemask, Delaire or Petit, mainly applies stress to the chin cap. The stress was more intense in the chin component of the Petit model as a reflection of its lower surface area. In the model by Gazzani et al. [25], stress distribution was calculated for an applied load of 9.8 N, which is equivalent to 1 Kg. However, about 18% of the practitioners in the present survey reported applying an even heavier force to the facemask through the elastics. Gazzani et al. [25] also stated that the supports should fit the patient’s face in the best way to maximize the contact surface with the skin for a more homogeneous distribution of the applied load and to avoid skin wounds.

Kim et al. [16] estimated that a facemask customized to the patient’s anatomy based on a 3D scan of the face and manufactured through 3D printing of the individualized components [26] would increase fit and comfort and decrease skin irritation.

The advantages of customization would be particularly evident in the youngest children, for whom the marketed standard facemasks may result in being largely oversized. In this regard, it is interesting to note that 80% of the surveyed orthodontists typically started facemask treatment at an age younger than 8 years.

The analysis by Gazzani et al. [25] also implied a greater risk of gingival recessions of the lower incisors for the Delaire type of facemask, as an effect of stress concentration at the upper border of the chin cap that may transmit to the labiomental sulcus area. It is remarkable that in the present survey the onset of gingival recession during facemask treatment was rarely noted by almost half of the respondents. As part of the custom modeling of the chin cap, the upper border can be designed away from the labiomental groove and made to extend more laterally on the mandibular body, where the gingival margins of the teeth run at a more cranial level.

A greater extension of the chin support sideways may also aid facemask stability during sleep, thus limiting the occurrence of dislocations at night that are reported by parents to be frequent with currently used standard devices.

The present survey also pointed out that orthodontists also ask the patients to wear the facemask at daytime for a varying number of hours, and they agree that the opportunity to objectively assess wear time would be advantageous for the good outcome of the therapy. Temperature microsensors embedded in the marketed facemasks to record wear time have previously been tested [13,21,22]. The consistent finding in these studies was that patients wore the appliance less than requested by the orthodontists. In particular, Arreghini et al. [21] concluded that monitoring per se did not effectively incentivize compliance.

Only a minority of SIDO members suggested to the parents ways to present the therapy to the child as a game or to reward the child for good compliance. It would be interesting, therefore, to verify whether a gamification approach to the therapy, implying a reward mechanism to stimulate the child’s cooperation, could also be an advantageous strategy in facemask treatment. As a part of the facemask-customization process, it has been possible to integrate in the forehead rest a temperature sensor that transfers the application-hours data to a docking station for wear-time monitoring by the doctor, as well as to a game application for smartphone or tablet, which is designed to reward a child’s good compliance [27].

The difference in the level of specialization did not reflect significant differences in most aspects of the clinical management of facemasks. The differences between S and GD highlighted by this survey were with regard to the type of facemask provided to patients and the most critical period of treatment. The majority of GD used the Delaire facemask whereas the majority of S used the Petit facemask. Lee et al. [10], when comparing the treatment effects produced by the two types of facemasks, concluded that no significant differences were present for most skeletal and dental changes. It seems, therefore, that the choice of either one of the two designs can be left to the clinician’s preference.

S and GD also differed significantly in their judgment of the most critical time of treatment, which for the majority of GD was the initial period whereas for S was the final period. Actually, both periods represent potential critical times for patient’s compliance during therapy for different reasons. The initial period can be critical due to the initial adaptation to the appliance whereas the final period can be critical because the patient is tired of wearing the mask.

A limitation of the present survey was the low response rate, which was probably due to the fact that it was performed immediately after the COVID-19 lockdown when the motivation to participate in an online survey was probably very low. Additional limitations were that we did not investigate the experience levels of the participating orthodontists, or how frequently these participants oversaw their patients during treatment. We also did not investigate whether the practitioners involved were adequately trained in employing facemasks. Future similar surveys should also take into account other important aspects like the functional assessment/re-education of patients with breathing or swallowing problems and postural therapy.

## 5. Conclusions

This survey showed the following:

- The facemask is not well received by the patients who, along with their parents, express concerns regarding its tolerability even before the start of treatment.
- Bulkiness and poor adaptation of the standard facemasks are often reflected in instability of the devices and in the frequent onset of skin irritations, especially on the chin. Such complications may disrupt the continuity of treatment and greatly discourage a patient’s cooperation.

## Figures and Tables

**Table 1:** Questionnaire administered to SIDO members. Frequency and percentage are reported for qualitative answers. Median and interquartile interval are reported for Likert scales. An asterisk (*) after the question indicates that there can be more than one answer.

1. Do you agree to participate?		
I agree and provide my informed consent		
2. Are you a Specialist in Orthodontics?		
No 61	(40%)	
Yes 90	(60%)	
3. To what age range do patients you provide facemask therapy for Class III malocclusion most frequently belong to?		
Earlier than 5 years	1	(1%)
Between 5 and 8 years	120	(79%)
After 8 years	30	(20%)
4. What type of facemask do you most frequently provide to your patients?		
Delaire facemask	78	(52%)
Petit facemask	73	(48%)
Grummons facemask	0	(0%)
5. How long in the 24 h-interval do you request your patients to wear the facemask? *		
In the afternoon and at night	72	
At night	32	
As long as possible when at home	51	
Other (specify)	9	
6. Usually, for how many months do you request the patient to wear the facemask? *		
6 months	24	
9 months	62	
12 months	54	
Other (specify)	19	
7. How much force do you apply through the elastic bands on each side of the facemask?		
Less than 500 g	34 (22%)	
500 g	90 (60%)	
More than 500 g	27 (18%)	
8. At the time of diagnosis, how do parents react to the proposal of facemask therapy for their child? Please indicate on a scale from 1 (Never) to 5 (Always) how often the following statements occur.		
Median (interquartile interval).		
They are perplexed	3 (2; 4)	
They are doubtful	3 (2; 3)	
They think it will be easy to adhere to the therapy	3 (2; 4)	
They think their child will accept the therapy	4 (3; 4)	
They think that wearing the facemask will be painful	3 (2; 3)	
They think that the therapy will give the desired results	4 (4; 4)	
9. What questions do you most frequently receive from parents regarding facemask treatment? Yes (%) *		
Can the facemask be worn at night?	42 (28%)	
How many hours per day does the child have to wear the facemask?	96 (64%)	
For how long will the facemask treatment last?	108 (72%)	
Does the facemask cause pain?	63 (42%)	
Should the facemask be worn at school too?	48 (32%)	
Can the child eat and drink while wearing the facemask?	16 (11%)	
Can the child sleep with the facemask on?	93 (62%)	
Can the child play sports with the facemask on?	16 (11%)	
Can the facemask be worn together with glasses?	23 (15%)	
How should the facemask be cleaned?	30 (20%)	
Other (specify)	2 (1%)	
10. Among those listed above, what questions do you most frequently receive directly from the patients? Yes (%)		
Can the facemask be worn at night?	14 (9%)	
How many hours a day do I have to wear the facemask?	53 (35%)	
For how long will the facemask treatment last?	80 (53%)	
Does the facemask cause pain?	62 (41%)	
Should the facemask be worn at school too?	59 (39%)	
Can I eat and drink while wearing the facemask?	21 (14%)	
Can I sleep with the facemask on?	44 (29%)	
Can I play sports with the facemask on?	24 (16%)	
Can the facemask be worn together with glasses?	11 (7%)	
How should the facemask be cleaned?	3 (2%)	
Other (specify)	1 (1%)	
11. What are the most critical times during facemask treatment?		
The initial period of treatment, when the patient may have trouble tolerating the new device or adapting to it.	82 (54%)	
The final months of treatment, when the patient may demonstrate disaffection with the therapy and/or lack of motivation for it.	60 (40%)	
Other (initial and final)	9 (6%)	
12. What problems do parents most frequently report during facemask therapy? Indicate on a scale from 1 (Never) to 5 (Always) how often parents report the following problems. Median (interquartile interval).		
Skin irritations	4 (3; 4)	
Lesions of the mucosae at the mouth corner or in the lower lip	2 (2; 3)	
Pain	2 (1; 2)	
Discomfort	3 (2; 4)	
Rigidity of the material of forehead and chin supports	2 (1; 3)	
Poor aesthetics	2 (1; 4)	
Shame	2 (1; 3)	
Difficulties in child falling asleep	2 (2; 3)	
Difficulties in motivating the child	3 (2; 3)	
Nocturnal awakenings	2 (1; 3)	
Difficulties in applying the facemask	2 (1; 2)	
Hygiene problems	2 (1; 2)	
Facemask dislocation during sleep	3 (2; 3)	
13. What problems do patients most frequently report during facemask therapy? Indicate on a scale from 1 (Never) to 5 (Always) how often patients report the following problems. Median (interquartile interval).		
Skin irritations	3 (3, 4)	
Lesions of the mucosae at the mouth corner or in the lower lip	2 (1, 3)	
Pain	2 (1, 3)	
Discomfort	3 (2, 4)	
Rigidity of the material of forehead and chin supports	1 (1, 2)	
Poor aesthetics	2 (1, 3)	
Shame	2 (1, 3)	
Difficulties in child falling asleep	2 (1, 3)	
Lack of motivation to wear the facemask	3 (2, 3)	
Nocturnal awakenings	2 (1, 3)	
Difficulties in applying the facemask	2 (1, 2)	
Hygiene problems	2 (1, 2)	
Facemask dislocation during sleep	3 (2, 3)	
14. Have you noticed the development of gingival recessions of the lower incisors in patients treated with the facemask, due to pressure by the chin rest? Indicate on a scale from 1 (Never) to 5 (Always) how often patients report the problem. Median (Interquartile interval).		
	1 (1, 2)	
15. Sort by severity the factors that hinder the success of treatment.		
(1 the least serious—8 the most serious) Rank for each factor. Median (interquartile interval).		
1. Patient compliance	7 (2, 8)	
2. Parents collaboration	6 (2, 7)	
3. Treatment duration	4 (3, 6)	
4. Poor adaptation of the facemask to the anatomy of the face	4 (3, 5)	
5. Aesthetics of the facemask	4 (2, 6)	
6. Rigidity of the material of forehead and chin supports	4 (2, 5)	
7. Difficulties in customizing the facemask	4 (3, 7)	
8. Inability to monitor the facemask wear time and the adequacy of the applied force on a daily basis	5 (3, 6)	
16. How do you stimulate the patient to be compliant? *		
I rely on the parents’ ability to motivate the child	12 (8%)	
I explain the parents what the therapy consists of and what results are expected, thereby motivating them to obtain or stimulate the child compliance	142 (94%)	
I present the facemask therapy as a game	44 (29%)	
I provide the child prizes and rewards for good compliance	7 (5%)	
I suggest the parents to provide the child prizes and rewards for good compliance	11 (7%)	
Other (specify)	8 (5%)	

**Table 2:** Comparisons between General Dentists practicing Orthodontics (GD) and Specialists in Orthodontics (S) for questions 3 through 7.

Variable	GD N = 61	S N = 90	*p*-Value FET
Q3 Age ≤ 8 years	48 (79%)	73 (81%)	0.8357
Q4 Delaire facemask	42 (69%)	36 (40%)	0.0005
Q4 Petit facemask	19 (31%)	54 (60%)	0.0005
Q5 To wear as long as possible	18 (30%)	33 (37%)	0.3859
Q6 To wear ≥ 9 months	55 (90%)	73 (81%)	0.1675
Q7 Force ≥ 500 g	45 (74%)	72 (80%)	0.4287

FET: Fisher’s Exact Test.

**Table 3:** Comparisons between General Dentists practicing Orthodontics (GD) and Specialists in Orthodontics (S) for question 11 (“What are the most critical times during facemask treatment?”).

Period	GD N = 61	S N = 90	*p*-Value χ^2^ Test
Initial	40 (66%)	42 (47%)	0.0188
Final	16 (26%)	44 (49%)	
Initial and final	5 (8%)	4 (4%)	

**Table 4:** Comparisons between General Dentists practicing Orthodontics (GD) and Specialists in Orthodontics (S) for question 14: “Have you noticed the development of gingival recessions of the lower incisors in patients treated with the facemask, due to pressure by the chin rest? Indicate on a scale from 1 (Never) to 5 (Always) how often patients report the problem.” Results reported as median (interquartile interval).

Question 14	GD N = 61	S N = 90	*p*-Value Mann–Whitney Test
Frequency of gingival recession	1 (1; 2)	2 (1; 2)	0.0581

**Table 5:** Comparisons between General Dentists practicing Orthodontics (GD) and Specialists in Orthodontics (S) for question 15: “Sort by severity the factors that hinder the success of treatment. (1 the least serious—8 the most serious). Rank for each factor.” Results reported as median (interquartile interval).

Factor	GD N = 61	S N = 90	*p*-Value Mann–Whitney Test
Patient compliance	7 (2; 8)	7 (2; 8)	0.5145
Parents collaboration	6 (3; 7)	6 (2; 7)	0.7402
Treatment duration	4 (3; 6)	5 (3; 6)	0.4702
Poor adaptation of the facemask to the anatomy of the face	4 (3; 5)	4 (3; 5)	0.6445
Aesthetics of the facemask	4 (1; 5)	4 (2; 6)	0.5069
Rigidity of the material of forehead and chin supports	5 (2; 5)	4 (2; 6)	0.6455
Difficulties in customizing the facemask	4 (2; 6)	4.5 (3; 7)	0.0756
Inability to monitor the facemask wear time and the adequacy of the applied force on a daily basis	6 (3.5; 8)	5 (3; 6)	0.1932

**Table 6:** Comparisons between General Dentists practicing Orthodontics (GD) and Specialists in Orthodontics (S) for question 16. “How do you stimulate the patient to be compliant?”; yes (%).

Answer	GD N = 61	S N = 90	*p*-Value FET
I rely on the parents ability to motivate the child	6 (10%)	6 (7%)	0.5467
I explain the parents what the therapy consists of and what results are expected, thereby motivating them to obtain or stimulate the child compliance	57 (93%)	85 (94%)	1.0
I present the facemask therapy as a game	18 (30%)	26 (29%)	1.0
I provide the child prizes and rewards for good compliance	5 (8%)	2 (2%)	0.1191
I suggest the parents to provide the child prizes and rewards for good compliance	4 (7%)	7 (8%)	1.0

FET: Fisher’s Exact Test.

## Data Availability

The data will be available on request.
